# Genetics as a key to improving crop photosynthesis

**DOI:** 10.1093/jxb/erac076

**Published:** 2022-03-02

**Authors:** Tom P J M Theeuwen, Louise L Logie, Jeremy Harbinson, Mark G M Aarts

**Affiliations:** 1 Laboratory of Genetics, Wageningen University & Research, Wageningen, The Netherlands; 2 Biophysics, Wageningen University & Research, Wageningen, The Netherlands; 3 University of Cambridge, UK

**Keywords:** Bi- and multiparental populations, diversity panels, gene validation, genome wide association studies (GWAS), improving photosynthesis, natural genetic variation, quantitative trait locus (QTL) mapping

## Abstract

Since the basic biochemical mechanisms of photosynthesis are remarkably conserved among plant species, genetic modification approaches have so far been the main route to improve the photosynthetic performance of crops. Yet, phenotypic variation observed in wild species and between varieties of crop species implies there is standing natural genetic variation for photosynthesis, offering a largely unexplored resource to use for breeding crops with improved photosynthesis and higher yields. The reason this has not yet been explored is that the variation probably involves thousands of genes, each contributing only a little to photosynthesis, making them hard to identify without proper phenotyping and genetic tools. This is changing, though, and increasingly studies report on quantitative trait loci for photosynthetic phenotypes. So far, hardly any of these quantitative trait loci have been used in marker assisted breeding or genomic selection approaches to improve crop photosynthesis and yield, and hardly ever have the underlying causal genes been identified. We propose to take the genetics of photosynthesis to a higher level, and identify the genes and alleles nature has used for millions of years to tune photosynthesis to be in line with local environmental conditions. We will need to determine the physiological function of the genes and alleles, and design novel strategies to use this knowledge to improve crop photosynthesis through conventional plant breeding, based on readily available crop plant germplasm. In this work, we present and discuss the genetic methods needed to reveal natural genetic variation, and elaborate on how to apply this to improve crop photosynthesis.

## Teaser

Natural genetic variation is an undervalued resource in the study of photosynthetic functioning. Quantitative and molecular genetics can be used to exploit natural genetic variation for improving photosynthetic performance of crops.

## Introduction

Plants are largely sessile organisms that depend on their genetic composition to survive in a given environment. The environment will normally change between geographic locations and over time. When species evolve, selection pressures from these environments (re)shape their genomes by incorporating genetic variations that improve their fitness. Approximately 1.5 billion years ago a cyanobacterium was engulfed by a phagotrophic eukaryote (Löffelhardt, 2014). This unique symbiotic incident eventually led to the transformation of the cyanobacterium into a chloroplast, and formed the basis for the evolution of plants. It brought together three genomes, the nuclear, the mitochondrial, and the plastidial genomes. Coevolution between these shaped photosynthesis while plants spread to all corners of the globe. The evolution of, for example, carbon concentrating mechanisms to overcome high photorespiration rates in C_3_ photosynthesis, and the occurrence of chlorophyll *d* in some cyanobacteria to make better use of the light spectrum available are a testament to the adaptability of photosynthesis (Keeley and Niyogi, 2003; Gloag *et al.*, 2007). Nowadays, with so many plant species occupying a wide diversity of niches and dynamic environments, differences in photosynthetic performance due to natural genetic variation occur for a range of processes, such as metabolism, growth, and responsiveness to environmental cues (Björkman and Holmgren, 1963; Flood *et al.* 2011; Yamori *et al.*, 2014; Soleh *et al*., 2016, 2017; Arrivault *et al.*, 2019; Rungrat *et al.*, 2019; Faralli and Lawson, 2020; Acevedo-Siaca *et al.*, 2020).

Despite this variation in photosynthetic performance, the basic biochemical mechanisms of photosynthesis have remained remarkably conserved within C_3_, the different forms of C_4_, and CAM plants (Bungard *et al*., 1999; Flood *et al.*, 2011). These basic biochemical mechanisms take place in the core photosynthetic machinery, and are encoded by around a hundred genes (Tyagi and Gaur, 2003; Berry *et al.*, 2013). Here we define the core photosynthetic machinery as the enzymes and the multi-molecular complexes required for the light and dark reactions of photosynthesis. The tight interaction within the multi-molecular complexes is likely to limit evolution of the core photosynthetic mechanisms (Shi *et al.*, 2005). Consequently, the observed photosynthetic variation between species (e.g. Wullschleger, 1993) and within species (e.g. Driever *et al.*, 2014; Prinzenberg *et al.*, 2018; Taniyoshi *et al.*, 2020; Faralli and Lawson, 2020; Acevedo-Siaca *et al*., 2020, 2021*a*; McAusland *et al.*, 2020) is most prominent in the remaining 3000 genes, whose coordinated action mediates photosynthesis (P. Wang *et al.*, 2017). It should be noted that in some cases photosynthetic variation is explained by variation in the abundance of core photosynthetic components—while maintaining the original function (Yin *et al.*, 2010; Kasajima *et al.*, 2011; Chao *et al.*, 2014; Simkin *et al.*, 2019; Rungrat *et al.*, 2019). Furthermore, in line with the omnigenic model, which argues that essentially any gene expressed in a tissue will be in some way involved in the complex phenotype of that tissue (Boyle *et al.*, 2017), many more genes are required for general functioning of a plant to ensure that photosynthesis can occur. The notion that natural genetic variation for photosynthetic functioning occurs primarily outside the basic biochemical mechanisms is further supported by studies in which phenotypic variation in photosynthetic traits is linked to the underlying genetic variation. The absence of core photosynthetic components amongst these genes confirms that variation in photosynthesis lies primarily outside the core photosynthetic processes (e.g. Q. Wang *et al.*, 2017; Van Rooijen *et al.*, 2017; Oakley *et al.*, 2018; Rungrat *et al.*, 2019; Adachi *et al.*, 2019; Prinzenberg *et al.*, 2020). With few genes identified so far in genetic studies, we do not state there is no functional natural genetic variation in core photosynthetic components, but the evidence points to the thousands of genes outside the core photosynthetic components as holding most of the genetic variation underlying variation in photosynthesis.

A lot of what we know now about the genetics of photosynthesis is based on the studies of induced mutations in *Chlamydomonas reinhardtii* (Levine, 1968) and Arabidopsis (Scheller *et al.*, 2001; Alonso *et al.*, 2003). The identified photosynthetic mutants were essential to assign biochemical functions to the corresponding genes and proteins (Rochaix, 2004). While mutants selected upon induced mutagenesis often display a drastic and obvious phenotype, as they are frequently caused by loss-of-function, or ‘knock-out’, mutations that disrupt gene function completely, natural phenotypic variation is generally much more subtle than the phenotypic variation seen in selected mutants. The drastic phenotype of knock-out mutants hardly ever allows the plants to survive the dynamic conditions encountered in the field. Natural genetic variation rarely involves knock-out mutations, but rather mutations that modify the function of the gene, often only slightly, leading to fitness enhancing, rather than disrupting, phenotypic changes.

**Fig. 1.**
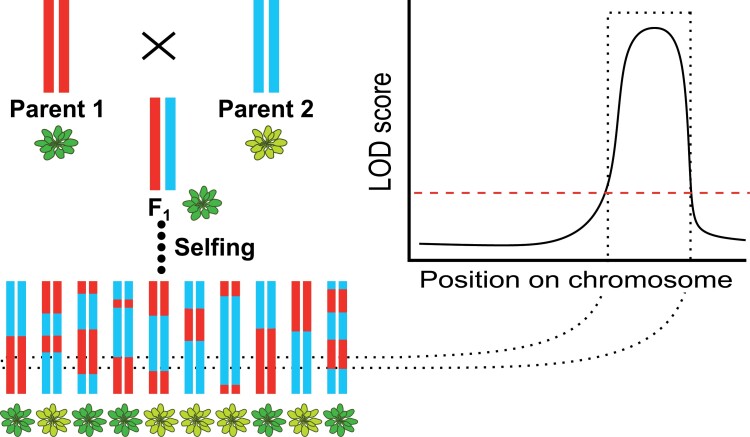
Concept of QTL mapping. The example illustrates how a recombinant inbred line (RIL) population is used to correlate genetic variation (depicted in blue and red) and phenotypic variation (depicted in light green and dark green). The likelihood of the association is given as logarithm of odds (LOD) score, where higher values point to stronger associations. The regions on the genome, a locus, with a LOD score above the multiple-testing-corrected threshold is termed a QTL. The principle shown here for a RIL population can be used in all types of bi- and multiparental and GWA mapping approaches.

The main route to explore natural genetic variation is using genetic mapping approaches to reveal quantitative trait loci (QTLs) underlying phenotypic differences in photosynthetic processes (Box 1; Fig. 1). Besides the already mentioned studies on linking genetic variation to photosynthetic variation, there is a growing body of literature on mapping studies in many plant species (e.g. Jung and Niyogi, 2009; Lowry *et al.*, 2013; Chao *et al.*, 2014; Ortiz *et al.*, 2017; Feldman *et al.*, 2018; Joynson *et al.*, 2021; Ferguson *et al.*, 2021). These QTLs may be selected in marker assisted breeding for improved photosynthesis, where information on the candidate genes is not essential to improve the trait. However, identifying a QTL will not provide much information on the biological mechanism responsible for the phenotypic differences caused by the QTL, which would be essential in expanding our knowledge on photosynthesis. While many QTLs for photosynthetic phenotypes are known, each QTL typically covers a genomic region containing dozens of candidate genes (Box 1), and only rarely have the causal genes, and the allelic DNA sequence variation, been functionally validated (Bernardo, 2016). If we want to know more about the role of standing genetic variation for photosynthesis in plant growth and crop yield, the causal genes need to be identified, and the allelic variation for these genes needs to be studied to understand how it causes phenotypic differences. In this review we discuss why photosynthesis is rarely maximal in nature and difficult to improve through plant breeding without detailed understanding on genotype–phenotype relations. We also show how genetics can be used to reveal standing genetic variation for photosynthetic traits and to learn more about photosynthetic regulation, and how this may be applied to improve the photosynthetic performance of crops, and ultimately crop yield.

**Box 1. The concept of genetic mapping**

To reveal genetic variation for a given phenotype, it is essential to correlate phenotypic variation to causal genotypic variation. For decades this has been done by genetic linkage mapping approaches (Nordborg and Weigel, 2008). A linkage map represents the order of and distance between genetic markers, based on the recombination frequency between the markers, for each chromosome. Recombination frequencies are best determined in genetically segregating populations, often progeny of a cross between two genetically distinct parents. When such parents are diploid and inbred, and thus homozygous, there will at most be two different alleles per marker or gene. Alleles are sequence variants of the same stretch of DNA, which can comprise a gene or part of a gene, but can also correspond to non-coding DNA. Alleles can only be distinguished based on DNA sequence differences, such as a single nucleotide polymorphism (SNP), an insertion or deletion (InDel), or another kind of sequence variation. Different alleles may confer the same plant phenotype. In mapping approaches the variation for a particular trait, say chlorophyll content, will be determined and correlated with variation between the marker alleles at each genetic locus. If a genetic locus contributes to the trait, this will be due to an allelic difference in one of the genes residing at that locus, meaning one allele contributing to below average chlorophyll content, the other allele contributing to above average chlorophyll content (Fig. 1). Often the marker is not a genetic sequence variant of the gene involved, but genetically closely linked to it. This means, the marker identifies a genetic locus, as it resides in the vicinity of a gene for which each of the two parents of the segregating population carries a different allele, one contributing to low chlorophyll content, the other to high chlorophyll content. Since chlorophyll content is a quantitative trait, expressed in values rather than a classification, such a locus is generally referred to as a quantitative trait locus, or QTL. The likelihood of the association between a marker and the phenotype depends on the mean effect size difference between the allelic groups and the standard deviation around the mean of the phenotype (Xu, 1995). Consequently, QTLs for phenotypes with larger effects and smaller standard deviations will be mapped with more confidence than those for phenotypes with smaller effects or larger standard deviations. Likewise, if a trait is highly polygenic and affected by many genes with different alleles in the two parents, the effect size attributable to each gene is smaller than where the trait is only affected by one variant gene, and the QTLs are harder to map (Korte *et al.*, 2013).

Often biparental mapping approaches are employed in linkage mapping that can use different types of segregating populations. This could be single-use F_2_ populations, or it could be ‘immortal’ populations of recombinant inbred lines (RILs), near-isogenic line (NILs), back-cross inbred lines (BILs), or doubled haploid (DH) lines (Bazakos *et al.*, 2017). RILs are constructed via repeated selfing of F_2_ progeny of one F_1_ hybrid, propagated through single-seed-descent. NILs and BILS are constructed via recurrent backcrossing of F_1_ or F_2_ progeny to one of the parents, and DHs are constructed through microspore culture or parental genome elimination in the F_1_, and subsequent (spontaneous or induced) doubling of the haploid genome (Bazakos *et al.*, 2017). Besides biparental populations, multi-parent advanced generation inter-cross (MAGIC) populations may be used, derived from crosses between different F_1_ progeny with more than two parental genotypes (Beyer *et al.*, 2008). While linkage mapping provides strong statistical power to identify a QTL, it does not provide a high resolution as to which allelic difference causes a QTL. A higher resolution can be achieved with subsequent fine-mapping so as to identify sufficiently small regions of the genome holding only one or a few genes associated with the QTL (Jaganathan *et al.*, 2020).

The increasing ease of generating whole genome sequences has made higher resolution physical maps available, based on a large set of densely spaced sequence markers, often SNPs or InDels (Zargar *et al.*, 2015). Diversity panels consisting of different, not obviously related, genotypes, representing many different recombination events are increasingly used to provide marker-dense maps to identify QTLs (van Bezouw *et al.*, 2019). They often consist of different (natural) accessions of a (wild) species collected at different geographic locations and different niches, but can also be composed of different varieties or breeding lines of a crop species. Such populations are especially attractive for genome wide association studies (GWAS), in which phenotypes are tested for association with small genomic regions, due to the high frequency of recombination events in the population. QTLs identified this way are far narrower than those identified upon linkage mapping in a biparental population, and thus contain far fewer candidates for the causal gene, though the confidence to map such loci is lower than for QTLs identified in biparental populations (Nordborg and Weigel, 2008).

## Improving the photosynthetic performance of crops

### Can photosynthesis be improved?

While there is plenty of natural and induced genetic variation for photosynthetic functioning, the question arises whether photosynthetic performance can actually be improved by breeding, using either conventional or novel plant breeding techniques. The term ‘improving’ is very broad, and depending on the perspective it may have different meanings (Zelitch, 1975). In this context we refer to improving photosynthetic performance, as either capturing more light energy or using each unit of absorbed light energy to more efficiently fix CO_2_, under some set of environmental conditions, relative to what is currently possible with a reference genotype or an elite cultivar. The ‘improvement’ may be realized at any of the levels of organization at which photosynthesis is normally measured (e.g. enzyme, protein complex, thylakoid, chloroplast, leaf, and canopy), but in an agricultural context it will be important that the improvement can be seen to act at the level of the canopy or field. With the global human population increasing and the climate changing rapidly, the need for agriculture to constantly push the limits of crop production is likely to increase.

Crop yield can be understood in terms of the efficiency indices: (i) light energy interception by the canopy, (ii) the conversion of that absorbed light energy into the chemical energy of biomass, and (iii) the harvest index (Monteith, 1994; Long *et al.*, 2006). Yield increases following the Green Revolution were remarkable and largely inspired by increases in the harvest index and light interception index of crops. By further improving these two efficiency indices, plant breeding contributes to an annual increase in crop productivity. However, this increase is levelling off for the most important staple crops, making it very challenging to meet the increasing demand for higher crop yield (Zhu *et al.*, 2010; Ray *et al.*, 2012). The third index contributing to crop yield, describing the efficiency of the conversion of intercepted radiation into the chemical energy of biomass, is largely determined by photosynthesis and respiration (Monteith, 1994; Long *et al.*, 2006). To illustrate the scope of improvement, for soybean, a C_3_ species, the current efficiency of intercepted radiation is such that 1.5% of the full spectrum of solar energy per unit area is converted into biomass (Zhu *et al.*, 2010). The theoretical maximum calculated for C_3_ species is 4.6%, implying there is potential for a staggering 3-fold improvement in biomass production by improving photosynthesis (Zhu *et al.*, 2010).

### Why has natural selection not led to improved photosynthesis?

If energy conversion in photosynthesis is so important, then why has this not been maximized by natural selection and evolution? The answer to this is complex, but it is likely that photosynthesis has been optimized rather than maximized. Photosynthesis is clearly an important process for plants that brings with it the benefits of fixed carbon and energy, but it also brings with it costs; reconciling these costs with the benefits of the process in different environments results in different optimal combinations of photosynthetic properties. In a rather crude way this can be seen in the morphology of parasitic plants in which photosynthesis is no longer used. The parasitic orchid *Rhizanthella gardneri*, for example, has no above ground parts and even flowers underground. It (and many other parasitic plants) dispenses with the costs of the above ground architecture necessary to support and position its leaves so they can function well as light absorbers for photosynthesis and at the same time be supplied with water and be able to export assimilates. This also implies that understanding the optimization of photosynthesis is not a matter that can be understood by analysing just leaf-level photosynthesis, but needs to be understood at the whole plant or, as is the case in agriculture, the canopy level.

Moving onto a simpler (albeit more restrictive) view of photosynthesis and focusing on leaves, there are numerous parameters that can be used to define photosynthesis. One of the most important of these is the maximum rate of photosynthesis as this defines a boundary to the effectiveness of the process as a source of carbon and a means for storing energy. The maximum rate can be that of a leaf, a genotype, a species, a type of plant, etc. Surprisingly, we know of no thorough theoretical analysis of the maximum leaf-level photosynthetic rate since that of Nobel (1991). He estimated for C_3_ photosynthesis a maximum theoretical CO_2_ assimilation rate of 55 μmol m^−2^ s^−1^ at an irradiance 2000 μmol m^−2^ s^−1^. This compares favourably with the highest measured rate of C_3_ photosynthesis (see Box 2)  of about 60 μmol m^−2^ s^−1^. Not many plants, however, come even close to these very high rates of photosynthesis. Annual C_3_ crop plants, species that are normally considered to have high photosynthetic rates, have rates of 20–30 μmol m^−2^ s^−1^ (Nobel, 1991), and there is considerable variation in the maximum photosynthetic capacity between types of plants (e.g. Larcher, 1995). In addition to maximum photosynthetic capacity, there are other photosynthetic parameters that contribute to the photosynthetic properties of a plant, for example water use efficiency (e.g. van den Boogaard *et al.*, 1997), phosphorous use efficiency (e.g. Denton *et al.*, 2007), nitrogen use efficiency (e.g. Evans, 1989), light-limited quantum yield (largely on a C_3_–C_4_ axis) (e.g. Ehleringer and Pearcy, 1983), and responses to fluctuating light (e.g. Harbinson and Woodward, 1984). Photosynthesis therefore exhibits rich variation in its properties throughout the plant kingdom though this variation may not always be completely understood in terms of a process of optimization. There are some examples where the link between the costs and benefits of a photosynthetic property are more straightforward. Across species, leaf nitrogen is broadly related to maximum photosynthetic rate (e.g. Evans, 1989; Reich *et al.*, 1994) and within a species this relationship is much stronger (e.g. Evans, 1988; Makino *et al.*, 2003). More photosynthesis needs more nitrogen but many habitats are nitrogen limited, making higher photosynthetic rates more uneconomic, resulting in an optimum for photosynthesis that is less than the potential maximum rate of 60 μmol m^−2^ s^−1^. If a plant has an insufficiency of nitrogen, photosynthesis will be depressed relative to the maximum achieved under conditions of adequate nitrogen nutrition (Evans, 1988). So the maximum rate of photosynthesis actually varies a lot from leaf to leaf and in most cases needs to be seen as the result of an optimization process rather than as a biophysical limit.

**Box 2. Maximizing photosynthesis in nature**

There are examples in nature where optimal photosynthesis is close to maximal photosynthesis. For some species growing in a sunny, warm, nutritious environment with ample water, but little plant competition and low pathogen and herbivore exposure, it may be advantageous to maximize photosynthesis, especially if the growing season is (very) short. Such conditions occur, but are rare. Think about semi-deserts, with generally sparse vegetation, but occasionally sufficient rainfall to allow short-lived, abundant plant growth. Some of the species with the highest photosynthetic rates are found at such sites, such as the winter annual *Chylismia claviformis*, occurring in the dry semi-deserts of the southwestern part of North America. This species has one of the highest CO_2_ assimilation rates reported for C_3_ plants, exceeding 60 μmol m^−2^ s^−1^ (Mooney *et al.*, 1976). Another remarkable species is *Amaranthus palmeri*, also from North American deserts, which is reported to show assimilation rates exceeding 70 μmol m^−2^ s^−1^, at a leaf optimum of 42 °C (Ehleringer, 1983). These examples can serve as models to understand how maximal photosynthesis has been selected in nature, and as examples of what may be possible by breeding.

To place this into perspective, in natural environments the fittest genotypes are selected, where fittest is defined as the ability to reproduce best (Popper, 1959). Even though improved photosynthesis might result in increased growth or higher seed yield, this might not be advantageous in a natural setting. Biotic and abiotic stresses, rather than suboptimal photosynthesis, are likely to impose more limitations on plant growth and reproduction in the field. Therefore photosynthesis is unlikely to be a permanent limitation on growth, and selection for improved photosynthesis may only infrequently occur, providing a poor driver of evolution. Furthermore, once a species has evolved its physiology to support photosynthesis in a given environment, adapting to a new environment can prove challenging (Leimu and Fischer, 2008). While in some cases this happens naturally, in other cases the required natural genetic variation is not available. If local populations of a species are geographically dispersed, gene flow will be limited making exchange of adaptive solutions difficult. Adaptation may also require too many steps to be taken before an optimum is reached, and if none of the intermediate steps improves fitness, the chances of reaching the optimum will be very low (as described by fitness landscapes). In other words, the complex nature of photosynthesis, which brings with it interdependency between components of the system, limits the options for changing any part of the system, and as such a change might prove to be disruptive for the system as a whole. Consequently, photosynthetic capacity can be fixed at a level well below the maximal for a given environment.

### Why has plant breeding not led to improved photosynthesis?

While natural systems may rarely select for plants with maximum photosynthesis due to various environmental and genetic constraints, these constraints may be removed for crop species, as agricultural systems are fundamentally different from most natural environments. Environmental conditions impairing production are actively minimized by watering, fertilizing, weeding, and pest management of crops. Trade-offs that may exist in a natural condition, favouring optimal over maximal photosynthesis, may be irrelevant in agriculture. Since natural selection for optimal photosynthesis has resulted in genetic variation for photosynthetic traits in many species, such could be exploited to improve photosynthetic performance of crops in agriculture. Plant breeding can facilitate gene flow to interconnect the optima of fitness landscapes, and allow the best photosynthetic performance to be selected in a way that would be difficult to achieve in nature. Studies into historically released cultivars show that in some crops increases in photosynthetic rate have been made, but in other crops there is no sign of such increase (Table 1). So, plant breeding has occasionally contributed to improved photosynthesis, though in part unintentionally due to a correlation between yield and photosynthesis. Yet, the absence of widespread photosynthetic improvements in crops, and the presence of considerable variation in photosynthetic performance in elite cultivars, demonstrates that there is no simple correlation between yield and photosynthetic performance (e.g. Driever *et al.*, 2014). This is likely due to the complex interactions between different mechanisms that act as bottlenecks for improving photosynthetic performance indirectly via selection for higher yield. It is here where direct phenotyping of components of photosynthetic functioning can contribute to improved photosynthesis and to identification of the bottlenecks that currently prevent yield increases.

**Table 1.** Overview of photosynthetic improvements in historically released cultivars in four major crops

Crop	Range of released date cultivars used in study	Main finding on photosynthetic improvements	Reference
Rice	1882–1976	Photosynthetic rate increased in the first half of 20th century, but afterwards improvement was less pronounced	Sasaki and Ishii (1992)
Rice	1893–1991	Photosynthetic rate improved only in some cultivars, but overall the photosynthetic rate correlated poorly with biomass	Zhang and Kokubun (2004)
Rice	1966–1997	Maximum photosynthetic rate decreased until 1980, but recovered slightly afterwards	Hubbart *et al.* (2007)
Wheat	1981–2008	Photosynthetic rate increased, but after early 2000 improvement was less pronounced	Zheng *et al.* (2011)
Wheat	1958–2007	No increase in conversion efficiency	Sadras *et al.* (2012)
Wheat	1967–2010	Photosynthetic rate increased	Ding *et al.* (2020)
Maize	1931–~1990	No increase in conversion efficiency	Ding *et al.* (2005)
Soybean	1934–1992	Photosynthetic rate increased	Morrison *et al.* (1999)
Soybean	1951–2006	Photosynthetic rate increased	Jin *et al.* (2010)
Soybean	1923–2007	Conversion efficiency increased	Koester *et al.* (2014)
Soybean	1923–2007	Maximum photosynthetic capacity has not increased, but daily carbon gain has increased	Koester *et al.* (2016)

### Status quo in improving photosynthesis

To assess the bottlenecks that can be targeted for photosynthetic improvements, the energy losses in photosynthesis have been modelled (Zhu *et al.*, 2010). The current understanding of the biochemical function of especially the core of the photosynthetic components allows the pinpointing of mechanisms that form bottlenecks. Alleviating these bottlenecks can range from relatively simple solutions, such as bypassing photorespiration and increasing the recovery from the photoprotective state, to more complex solutions such as converting crops from C_3_ to C_4_ photosynthesis (Zhu *et al.*, 2010). These elegant advances, achieved through genetic modifications, have been shown to be very effective (Kromdijk *et al.*, 2016; Driever *et al.*, 2017; South *et al.*, 2019; Simkin *et al.*, 2019; López-Calcagno *et al.*, 2020), but can also be very challenging to achieve (Ermakova *et al.*, 2020). It is noteworthy that the effective improvements in recovery from the photoprotective state that were shown to result in increased yields in tobacco (Kromdijk *et al.*, 2016) were not reproducible in Arabidopsis (Garcia-Molina and Leister, 2020), meaning that it is not a one-size-fits-all solution. Nevertheless, these approaches underline that improvements can be made, and undoubtedly more of these developments will follow.

**Fig. 2.**
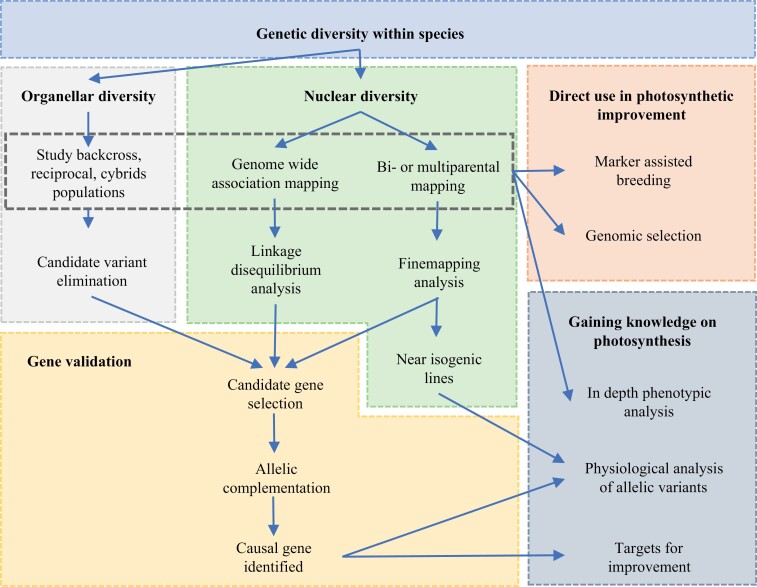
Schematic overview of how natural genetic variation within a species can contribute to improving photosynthesis. Through analysis of nuclear and organellar genetic diversity, interesting marker phenotype associations can be revealed. These can be used directly in marker assisted breeding and genomic selection programmes, and the populations can be used to study correlations and responses of photosynthetic phenotypes. In order to gain more knowledge on how natural genetic variation contributes to photosynthesis, the casual genes have to be identified via gene validation methods. Near isogenic lines and transformed lines containing different alleles can be used to deepen physiological processes in which these genes play a role. Ultimately some of these will form new targets for photosynthetic improvements.

So far most progress has been made in mechanisms that are part of the core photosynthetic apparatus, or processes directly linked to them. The absence of natural genetic variation in the core photosynthetic machinery makes genetic modification a suitable approach (Ort *et al.*, 2015). However, this dismisses the potentially thousands of other genes, for which there is natural genetic variation, that hold the capacity to improve photosynthesis. Moreover, genetic modification limits the range of crops that can be improved and the countries in which such crops can be grown. This is another reason to consider improving photosynthetic performance of crops via natural genetic variation as an attractive alternative. To improve photosynthetic performance through selection of standing genetic variation, two approaches can be followed, which are not mutually exclusive (Fig. 2). The most straightforward way is to exploit genetic variation for photosynthetic phenotypes in marker assisted breeding and genomic selection approaches of crops. This will of course need awareness of this option among plant breeders, as well as suitable germplasm, appropriate phenotyping facilities, and the right statistical framework, as support. A more sophisticated way would be to study the genes involved in photosynthesis that are not yet identified as such, and determine the genetic constraints underlying physiological bottlenecks in crop photosynthesis. With such knowledge, a systematic approach can be designed to improve crop photosynthesis and yield in a targeted way. This would open up knowledge on the thousands of genes involved in photosynthesis for which there is genetic variation, which so far have remained undiscovered and cannot be used to improve photosynthesis. To illustrate this, of the ~3000 Arabidopsis nuclear genes encoding a protein predicted to have a chloroplast target peptide, and to likely play a role in photosynthesis, only 15% have a known role in photosynthetic performance (Fristedt, 2017). Only for a very few of these genes has the phenotypic effect of allelic variation been explored.

## Quantitative and molecular genetics

So, what are the options on how quantitative and molecular genetics can help to reveal natural genetic variation for photosynthetic traits and establish the identity and function of the genes involved? To guide this discussion, a schematic overview is given in Fig. 2.

### Genetic mapping approaches to reveal nuclear genetic variation

Most plant traits are quantitative, meaning they are expressed in (non-discrete) values. Examples of quantitative traits are flowering time, plant biomass, and the efficiency of photosynthesis. The identification of genes underlying quantitative traits can be resolved by genetic mapping (Box 1). In order to decide on which mapping approach to use, it is important to discuss the advantages and disadvantages of the most common approaches. In the past, most genetic mapping involved bi- and multiparent mapping populations, but in the last decade, the use of diversity panels for genome wide association studies (GWAS) has gained importance (Bazakos *et al.*, 2017). While we will focus on discussing approaches for diploid inbreeding species, the concepts also apply to outbreeding and polyploid species.

Bi- and multiparental populations (Box 1) are very useful in revealing most of the heritability for a specific phenotype. The low genetic diversity in these populations, derived from only a few parental lines, contributes to a high statistical power to detect QTLs, due to the roughly equal distribution of alleles (e.g. 50:50 at each locus in a biparental population or 25:25:25:25 in a tetra-parental population). Though they require several generations to construct, once available they allow the use of replicate plants per genotype in the study, thereby providing high confidence of plant phenotypes. Immortal populations also provide the opportunity to use the same population for replicate experiments in different environmental conditions, allowing the identification of robust QTLs, expressed in several environments or over several years. Bi- and multiparental populations can also be effective in resolving non-additive genetic variation, such as that caused by epistatic interactions. Epistasis is a very common biological phenomenon, meaning that the phenotypic effect of an allele at a specific locus depends on the effect of an allele at another locus (Mackay, 2014). Due to  the interaction of these alleles, the statistical power to detect epistatic QTLs is lower than for additive QTLs, and sometimes insufficient, although always better for bi- and multiparental populations than for diversity panels used for GWAS (Nelson *et al.*, 2013; Mackay, 2014). Since photosynthesis is so complex, it is a prime example of a process in which epistatic interactions are likely to play a role. Thus, bi- and multiparental mapping holds great potential for revealing genetic variation for photosynthesis, especially when a large population can be screened, and even more so if the population is derived from phenotypically distinct parental lines, previously identified in a diversity screen for GWAS.

GWAS make use of diversity panels, rather than bi- or multiparent populations, which means a GWAS population, or diversity panel, typically represents much more genetic diversity and a higher density of recombination events between genomic regions than present in bi- or multiparental populations. The genomic region in which no recombination is found, when comparing all accessions in the diversity panel, is often referred to as being in linkage disequilibrium (LD) (Flint-Garcia *et al.*, 2003). In wild species, the decay of LD may be in the order of hundreds to a few thousands of base pairs, while in domesticated species, which have gone through a genetic bottleneck, the decay of LD can be in the range of 100-kbp (Flint-Garcia *et al.*, 2003). The high density of recombination events in wild species gives GWAS a very high mapping resolution (Bazakos *et al.*, 2017), and often restricts the QTL region to cover only a small number of candidate genes. Additionally, a diversity panel for GWAS is easily composed from available crop germplasm, or may be collected in a few months for wild species. If the species is autogamous, collected accessions may already be largely homozygous and nearly ‘immortal’, and thus may only need to be genotyped once. This has prompted GWAS as the upcoming approach to map genetic diversity in the past decade (Bazakos *et al.*, 2017). It was first established for Arabidopsis (Atwell *et al.*, 2010), but since then has been used for many more plant species (e.g. as reviewed in van Bezouw *et al.*, 2019; Gupta *et al.*, 2019; Hao *et al.*, 2020).

The high genetic variation of the diversity panels used in GWAS also means there may be several alleles for a locus, some of which may be represented at a high frequency in the panel, while others may be rare (Forsberg *et al.*, 2015). Alleles that are present at low frequencies are unsuitable for QTL analysis, as the statistical power to detect the QTL will be too low (Korte and Farlow, 2013). Adaptations to specific environments, as is often the case for photosynthesis-related traits, may result in high allele frequencies in local, adapted, populations, but when considered over the full diversity panel, comprised of accessions from many, global populations, the favoured allele may be rare, resulting in poor statistical power to detect it (Barboza *et al.*, 2013; Lopez-Arboleda *et al.*, 2021). Another important aspect to consider in GWAS is the effect of kinship in the ability to detect QTLs. If the diversity panel contains many accessions that are much more closely related to each other than to other (groups of) accessions, the population structure that results from this kinship may enhance the false detection of a QTL, as several markers will be shared among those related accessions, and a marker–phenotype association may occur just by chance. This means population structure must be accounted for, and can also statistically be corrected for, in GWAS, to avoid false associations to be detected as QTLs (Korte and Farlow, 2013). This does, however, mean that genetic variation that is tightly associated with a local lineage is difficult to detect, as the statistical correction ignores such associations. To get high resolution mapping, a very high genetic marker density will be needed, often in the range of hundreds of thousands or even millions of single nucleotide polymorphism (SNP) or insertion or deletion (InDel) markers. While this is now technically feasible (Jaganathan *et al.*, 2020), it poses problems with respect to the statistical analysis. With more markers to be tested for marker–phenotype associations, the chance of finding an association at random, without an actual causal relation between genetic variant and phenotype, will increase. This ‘multiple testing error’ can be corrected for by adjusting the significance threshold needed to classify a marker–trait association probability as significant. Most commonly used methods correct for multiple testing errors by taking a naïve Bonferroni or false-discovery rate threshold, or alternatively by performing permutation testing to take the underlying phenotypic distribution into account (Storey and Tibshirani, 2003; Freudenthal *et al.*, 2019, Preprint). QTLs identified with probability scores (often expressed as the minus logarithm of the *P*-value) above these thresholds are most likely to be valid QTLs that warrant further follow-up study. However, one should realize that QTLs that do not reach the significance threshold may still be valid, and QTLs that exceed the threshold may be false (Korte and Farlow, 2013). As a consequence of these issues, GWAS diversity panels often display large phenotypic diversity, and high heritability, but may only reveal a few QTLs. In most cases, QTLs with relatively low explained variance are detected, often barely reaching the significance threshold. This implies that while GWAS will reveal associations, the number of identified QTLs is often an under-representation of the total number of available QTLs present in the diversity panel. Especially when very few QTLs are found, one may want to resort to additional bi- and multiparental mapping approaches, using new or existing populations generated by crossing genotypes with interesting phenotypes as identified in the phenotypic screen that initiated the GWAS.

All of these genetic mapping methods, with their advantages and disadvantages, are commonly used in photosynthetic research. While somewhat variable between studies, biparental mapping (e.g. Jung and Niyogi, 2009; Yin *et al.*, 2010; Lowry *et al.*, 2013; Oakley *et al.*, 2018; Feldman *et al.*, 2018) seems equally successful in finding QTLs as GWAS (e.g. Chao *et al.*, 2014; P. Wang *et al.*, 2017; Ortiz *et al.*, 2017; Van Rooijen *et al.*, 2017; Rungrat *et al.*, 2019; Prinzenberg *et al.*, 2020; Joynson *et al.*, 2021; Ferguson *et al.*, 2021). Initially much of this work was performed in model species like Arabidopsis, for which suitable mapping populations are readily available, but these approaches are increasingly feasible in crop species. In order to develop immortal mapping populations as efficiently as possible, speed breeding allows for more generations per year, and fast construction of mapping populations in crops (Watson *et al.*, 2018). The costs of genotyping are still decreasing, and novel, cheaper genotyping approaches are still being developed (Gaio *et al.*, 2019, Preprint). Consequently, depending on the crop, and the time, budget, phenotype, and aim of the project, one should be conscientious in selecting the appropriate mapping approach.

### Marker assisted breeding and genomic selection

Once a QTL is identified as holding interesting genetic variation for a trait, it can feed directly into a marker assisted breeding programme to introduce and establish the favourable allele in elite crop lines, thereby improving photosynthesis. The success and impact of this will rely on the number of QTLs affecting the trait, and the percentage of phenotypic variance that is explained by the QTL. Photosynthesis is a highly polygenic trait, affected by many QTLs, often with relatively small effect sizes. Consequently, introgression of individual QTLs will be logistically complex, requiring large recombinant populations or many subsequent cycles of crossing and selection, to be able to select the rare recombinants that will combine several alleles with positive effects on the trait, while retaining all other important crop traits. Instead, genomic selection approaches might be more suited. Genomic selection attributes a weight to each individual marker, which depends on its association with the trait (Meuwissen *et al.*, 2001). So rather than identifying individual QTLs, it predicts the breeding value of genotypes based on the overall prediction of all markers combined, also known as genomic estimated breeding value for a genotype (Crossa *et al.*, 2017). In this way, the underlying function remains unknown, but in contrast to classical breeding where individual alleles have to be incorporated, in genomic selection approaches many alleles with a combined large effect on the trait can quickly be incorporated into elite breeding material. This has been shown to be very effective in yield improvements and disease resistance breeding (Beyene *et al.*, 2015; Rutkoski *et al.*, 2015), and is used in commercial breeding programmes for soybean and maize (Bernardo, 2016). Genomic selection has not yet contributed to improvements in photosynthetic performance, but this is likely to change when using natural genetic variation for improving photosynthesis becomes more mainstream. This will be especially the case if more becomes known on the interaction of photosynthesis QTLs with the environment, the so-called genotype×environment interactions. Photosynthesis phenotypes are prone to be affected by the environment (Murchie *et al.*, 2018), which means that alleles beneficial in certain crop production conditions may not be beneficial in another environment or agricultural system. Due to the speed at which genomic selection can proceed, different elite cultivars may rapidly be generated for a range of environments.

### Fine mapping and candidate gene validation

The identification of QTLs for photosynthetic traits in model and crop species has become more common in recent years, and QTLs can feed into marker assisted breeding programmes to improve photosynthesis. However, it is also relevant to study the function of the gene and alleles underlying the QTL, to better understand their role in photosynthesis (Fristedt, 2017). Revealing their function can increase the versatility of genetic modification studies and pinpoint relevant physiological mechanisms, to subsequently improve photosynthesis (Fig. 2). While, in principle, mutant screens are very effective for revealing the function of genes (Belcher *et al.*, 2015; Li *et al.*, 2019), a gene knock-out can be lethal, making it impossible to study the gene function via a knock-out mutant. Moreover, a knock-out mutant may show an altered photosynthetic phenotype, but such may be a pleiotropic effect, and only one aspect of a complex mutant phenotype, as the gene is only indirectly involved in photosynthesis. In such cases, it will remain difficult to determine the actual function of the gene with respect to photosynthesis. It is here that the study of allelic variation, in which functional differences convey more subtle differences, is useful. Knowledge on such subtle allelic variation is also much more likely to contribute to improving photosynthesis by breeding. As gene function analysis can be time consuming, such is especially feasible in model species like Arabidopsis, whereupon the knowledge can be translated to crops, or serve as an example for targeted follow-up studies in crops.

In revealing the responsible gene(s) underlying a QTL, a common mistake is to assume that the causal gene is the one closest to the marker with the highest genotype–phenotype association. Such is generally not the case, and proper gene validation is needed using a limited set of candidate genes. In the case of QTL mapping in bi- or multiparental mapping approaches, QTLs are often mapped to a large genomic region containing hundreds of genes. Resolving these will need one or more rounds of fine-mapping, using the generation of additional segregating populations, to reduce the QTL region by means of additional recombinations (e.g. Adachi *et al.*, 2019). Fine-mapping can benefit from the use of near isogenic lines (NILs), which vary for the QTL region, but are otherwise isogenic (Alonso-Blanco and Koornneef, 2000). Especially since photosynthesis is affected by many genes, by using NILs one will be able to examine the consequences of the variation in the  target region only, as there will be no genetic variation in  the rest of the genome. NILs can also be very useful to study the physiological impact of the QTL variation (e.g. Adachi *et al.*, 2014). To facilitate the speed in which NILs can be developed and used, especially for crops, it is worth considering the use of heterogeneous inbred families as a bi- or multiparental population, as these hold regions that are still heterozygous, allowing the quick identification of NILs (Tuinstra *et al.*, 1997). In GWAS, the mapping resolution is sometimes enough to directly pinpoint the causal gene, although this will require the availability of all sequence polymorphisms in all genotypes of the GWAS population, which is rare (Jaganathan *et al.*, 2020). Consequently, the marker is often simply a pointer to a region without any recombination in any of the genotypes in the studied population, also known as a haplotype block (Gabriel *et al.*, 2002). The decay of LD will determine the size of the haplotype block that carries the genetic variant responsible for the genotype–phenotype association. In principle all genes within this haplotype block could represent the variant allele causal for the target phenotype. Once the haplotype block is identified, it is tempting to determine possible causality of the obvious candidates in the region based on what is known about the predicted functions of genes in the block. One needs to be cautious though, as the function of many plant genes is still unknown, even for Arabidopsis (www.arabidopsis.org), because of which one may focus on a likely candidate and miss the actual causal gene, and thus the opportunity to shed further light on the biology underlying the target phenotype (Baxter, 2020). As further outlined below, the actual identification of the DNA sequence variant, be it a SNP, InDel, or other, is not trivial, is often laborious, and is sometimes unachievable, which may be frustrating.

When the number of candidate genes has been narrowed down to about 10, either upon fine-mapping in the biparental population, or upon LD analysis in GWAS, the gene validation can start. As a first step, and as a first step only, a mutant analysis of the remaining candidate genes is often the preferred next step. If a mutant with a loss-of-function allele produces a phenotype consistent with the function of the hypothetical wild-type gene giving rise to the phenotype used in the QTL analysis, then that mutant may point to the causal gene. Knock-out lines may be obtained via available stock centres, such as the T-DNA insertion lines for Arabidopsis (www.arabidopsis.info or abrc.osu.edu), or by CRISPR–Cas-mediated gene editing. As genotypes within a species can vary substantially for photosynthetic traits (Wójtowicz and Gieczewska, 2021), it is important to realize that the phenotype of a loss-of-function mutant may depend on the genetic background. Since most Arabidopsis T-DNA mutants have been generated in a Columbia (Col) background, there will not be any mutants for genes that are absent from Col or genes for which Col has a natural loss-of-function allele, both of which are common (Gan *et al.*, 2011). However, it is insufficient or even inappropriate to conclude on causality of the QTL by establishing that the phenotype of a knock-out allele of one of the candidate gene involves the same biological process that is studied in the QTL analysis. Note that with over 3000 genes involved in photosynthesis in Arabidopsis (which is about 1 in 10), there is a good chance that one of the genes of a haplotype block covering 10 genes will give a knock-out mutant photosynthesis phenotype. While this may indeed be the gene underlying the QTL, additional validation is needed to confirm this. Without such confirmation, one may have identified a gene that is somehow involved in photosynthesis, but not necessarily the gene underlying the QTL! A logical next step is to establish that there is genetic sequence variation between the alleles with contrasting phenotypes, to explain such phenotypic differences. Confirming variation in alleles may not be easy to do, as allelic variation may not be in the coding region, but in sequences regulating transcription. It may even be of an epigenetic nature, such as for the *FWA* gene, controlling flowering time in Arabidopsis (Soppe *et al.*, 2000).

Once there are only a few candidate genes remaining, meaning genes for which there is allelic variation between genotypes that may explain the phenotypic differences between the genotypes, and which have a function in line with the studied trait, the final step of the gene validation can start. In general two approaches are used to confirm the identity of the causal gene underlying a QTL. Of those two approaches transgenic complementation is most often used. This involves transforming the allelic variants into a loss-of-function mutant background, to recreate the phenotypic difference or variation initially used to identify the QTL (e.g. Alonso-Blanco *et al.*, 2005; Bentsink *et al.*, 2006; Loudet *et al.*, 2007). One will need to generate several independent transformation events, especially if the phenotype depends on the expression level of the introduced allele, which may be higher if more copies are introduced, and may depend on the site of T-DNA insertion. Transgenic complementation may be replaced by gene editing approaches, to substitute one allele for the other (Molla *et al.*, 2021), but this may not be feasible for all types of allelic variants. The alternative approach is to use quantitative allelic complementation (Weigel, 2012; Turner, 2014). This relies on crossing one or more accessions carrying one allele of the QTL with both a wild type and a knock-out mutant of the target gene, and do the same with one or more accessions carrying the alternative allele. If the target gene is not the one underlying the QTL, the phenotypic difference between F_1_s with wild-type or mutant plants will be similar for both allelic variants, while it will be different if the target gene is indeed the one for which allelic variation underlies the QTL. A case in which this has been successfully used was presented by Van Rooijen *et al.* (2017), showing the role of *YS1* in photosynthetic response to an increase in irradiance.

Once the causal gene is convincingly identified, the different alleles can be used to study the physiological role of the gene (Fig. 2) and learn more about its significance for photosynthesis and the potential to use it to improve photosynthesis, either through breeding or genetic modification. So while there are several approaches to gain new insights in physiological functioning of photosynthesis, in attempts to improve photosynthesis it will be best to be aware of the allelic variation and have a deeper understanding of the physiological functioning associated with this variation.

### Revealing organellar genetic variation

All of the methods described above focus on the exploitation of genetic variation in nuclear genomes, thus ignoring the genetic contribution from organellar genomes. Photosynthesis, however, is predominantly associated with processes occurring in the chloroplasts. The chloroplast holds roughly 70 protein-coding genes, most of which are essential for photosynthetic performance (Rochaix 1997). Also, mitochondria play a role in supporting photosynthesis (Nunes-Nesi *et al.*, 2008; Fan *et al.*, 2021), and they contain roughly 30 protein-coding genes. As organelles inherit mostly uniparentally, and recombination does not take place, beneficial alleles of organellar genes spread much less easily through the population of a plant species than alleles of nuclear genes. The uniparental inheritance also means that the study of natural genetic variation in these organelles through conventional mapping populations is much more difficult (Joseph *et al.*, 2013; Tang *et al.*, 2014).

Consequently, when studying genetic variation for photosynthetic traits, not only the nuclear genome, but also the organellar genomes and the nuclear–organellar interaction should be considered. This variation can be exploited for breeding purposes using recurrent backcrossing or the construction of cybrids, which are genotypes with novel combinations of nuclear and organellar genomes (Evans, 2007; Miclaus *et al.*, 2016; Roux *et al.*, 2016; Flood *et al.*, 2020; Lv *et al.*, 2020). Using cybrids it was conclusively revealed that phenotypic differences for photosynthetic traits can be caused by natural genetic variation in organelles (Flood *et al.*, 2020). As for the nuclear counterpart, it is also relevant to identify the causal organellar gene for a cytoplasmic trait. As fine-mapping is not possible in organellar genomes, cybrids with the same nuclear genome but different organellar genomes that differ in the candidate genetic variants can be used for gene identification. When the number of candidates is sufficiently low, organellar transformation methods can be used to reveal the casual gene. While recently many advances have been made, editing of chloroplast genes remains difficult (Molla *et al.*, 2021).

## From natural genetic variation to crop improvement of photosynthesis

In this review we have described how to identify natural genetic variation for photosynthetic traits, and perform validation of the genes and alleles involved. Photosynthesis is not an easy trait to study: it is highly polygenic and phenotypically highly responsive to environmental conditions (Zargar *et al.*, 2017; Kaiser *et al.*, 2018; Vico *et al.*, 2019). In crop production, environments are rarely constant, but typically highly dynamic, and inevitably, it will be challenging to reliably phenotype photosynthesis in dynamic conditions to support analysis of genetic variation (Soleh *et al*., 2016, 2017; Flood *et al.*, 2020; McAusland *et al.*, 2020; Acevedo-Siaca *et al.*, 2021*b*). It will be more the rule than the exception that apparently similar phenotypes are caused by different QTLs. In addition, there is likely to be a strong genotype by environment interaction, meaning that alleles that improve photosynthesis in one condition may be unfavourable in another. Adding to the challenge is that there is poor correlation between photosynthesis in constant and dynamic conditions (Acevedo-Siaca *et al.*, 2021*b*). Therefore, to allow the screening of functional variation for photosynthesis in dynamic conditions, it is essential to reproducibly mimic such conditions in controlled environmental facilities (as described by Murchie *et al.*, 2018) and to be able to phenotype photosynthesis at high throughput in such facilities, to reliably identify genetic variation amenable to breeding (van Bezouw *et al.*, 2019). At the moment this is possible for a range of photosynthetic parameters, but not for all (Siebers *et al.*, 2021). Especially the development of high-throughput phenotyping techniques to determine photosynthesis in controlled, but dynamic environments is required, as this will provide the reproducibility needed for genetic studies (e.g. Cruz *et al.*, 2016), and the opportunity to establish how to best breed for improved photosynthesis under field conditions. To assess the overall impact of the variation for a photosynthetic trait in a given environment, it is essential to use crop models to take into account the dynamic properties and broad ranges of environments (Wu *et al*., 2017, 2019; Coast *et al.*, 2021). Models will also be very useful to identify the potential contribution of photosynthesis improvements on yield, given the variation on other yield components, such as root nutrient uptake or sink capacity (Yin *et al.*, 2022). Using an approach where the impact of genetic variation on crop performance in a given agricultural context can be predicted will allow the identification of alleles that are deemed interesting to incorporate in breeding material. In the case of genomic selection procedures, the weight of an individual marker can be tested via crop models in the environment of interest, to steer the breeding for improved photosynthesis in a range of environments.

## Conclusion

Breeding for elite cultivars has largely ignored improvements in photosynthesis. While promising advances in improved photosynthesis may be achieved by genetic modification (Ort *et al.*, 2015; Hitchcock *et al.*, 2022), we argue that using natural genetic variation for photosynthesis holds an equally promising potential for improvement. While biotechnology applications largely focus on the core photosynthetic pathway, natural genetic variation will reveal the benefit of knowledge on the thousands of genes that ensure proper embedding of photosynthesis in plant metabolism and growth. Flood *et al.* (2011) already outlined natural genetic variation as a promising route, and indeed in the past decade an increasing number of studies have appeared revealing QTLs in different crops in a plethora of environments and photosynthetic traits. In the absence of a focused effort, largely caused by the complexity of the trait, it appears that few of these QTLs have been used to support breeding programmes, or even to gain an in-depth understanding of the physiological processes they could unveil. Natural plant species may not have maximized photosynthesis, as is desirable for high-yielding crops, but they evolved robust forms of photosynthesis that allow them to cope with many different, dynamic environments. In this sense, nature has taken millions of years to try new or alternative methods of converting sunlight, in both dynamic and broad-ranging environments. By identifying the bottlenecks in the photosynthetic function in these environments, and through targeted genetic studies on these traits, nature’s often elegant solutions to problems can be explored and learned from. As far as natural genetic variation for photosynthesis goes, we live in exciting times.
